# Experiments in Modelling Composition

**Published:** 1884-11

**Authors:** Stewart J. Spence

**Affiliations:** San Francisco


### SELECTIONS.

### Experiments on Modelling Composition.

BY STEWART J. SPENCE, D.D.S., SAN FRANCISCO.

In these experiments two kinds of the material were used, a hard and a soft. Four simple experiments were first made to test its elasticity, pliability, expansibility, and ductility, as follows:

A—*To test its elasticity.* Softened pieces in hot water, and rolled into ropes, each about an inch thick, and six inches long; holding one end down on the table, stretched the piece about an inch; each readily shrank back about one-sixth of an inch.

B—*To test its pliability.* Rolled it out as before, and placed an end of each piece projecting about two inches over the edge of a table. The soft readily bent down to the perpendicular, and the hard bent nearly so in a few minutes.

C—*To test its expansibility.* Rolled out a rope about an inch thick and twenty inches long, and while warm placed it on a table, each end touching a barrier. In half an hour it had shrank enough to admit a silver five cent piece at each end.

D—*To test its ductility.* Pressed the root of a tooth up through a mass of the material; the surface for some inches surrounding was raised thereby.

These primary tests being made, I proceeded to more complex experiments:

1st. A shallow flanged tray was filled with the hard composition, and a plaster model pressed into it a little distance, leaving exposed the upper labial and buccal surfaces, against which the material that had overflowed the tray was now pressed by the finger ; *as soon as the pressure was withdrawn the material withdrew also.* This is partly due to its pliability permitting it to yield to the force of gravitation, but chiefly to its elasticity: for when afterwards pressed downward away from the cast, it shrank back again *upward.* Thus we see that even in taking an impression of the inferior arch, the material may not be in contact with the gums, unless held so by the tray, or digital pressure. (It is not safe, I think, to rely on the latter.)

2nd. A similar test was made with the soft material, which fell away from the model very quickly. Its lack of rigidity and rather high specific gravity cause it to yield readily to gravitation.

3rd. The next experiment had reference to the effect of this last mentioned force on the impression of the palate. Hard composition was placed on the tray, and the plaster model pressed into it, the posterior border of the tray being brought within an eighth of an inch of the model, thus forcing out a mass of the material backwards, *after a few minutes the material had fallen away perceptibly from the palate of the model.*

4th. A similar test was made with the soft material, with increased bad results.

5th. A similar test was made, but varied in having a tray *which extended far back;* hard composition was used ; this time the falling away from the hard palate was not perceptible. The pliability of modelling composition is to be dreaded in removing impressions (especially when teeth remain), both in detaching the material from the tray, and in its drawing out of shape around the teeth.

6th. A cast of a lower full natural denture was pressed into soft composition in a lower tray, and on being withdrawn it detached the material from the tray, raising it about an eighth of an inch.

7th. Hard composition was more successful; but it yielded in a test.

8th. Where a cast was used on which a single bell-crowned tooth remained. *To avoid this the material should be attached to the tray by heating the tray over a flame.*

9th. An impression was taken of two teeth with a dove-tail space between them. After removing the impression, it would not readily fit back again, thus indicating that *the elasticity of the material adapts it for impressions of slight undercuts*, because it returns to some extent to its shape. But its pliability and ductility unfit it for partial cases where the undercut, or bell-crown, or dove-tail is considerable, because *on being removed it draws out of shape, not only the portion in immediate conjunction with the tooth, but also much of the surrounding surface.* This objection seems sufficient to contra-indicate it in most partial cases. Still if the hard material is used, and not withdrawn till thoroughly cold, this dragging is comparatively slight, as a moment's reflection will show, it being very ductile while soft but brittle when hard.

Allow me to digress to notice the theory that the dragging of wax, etc., in partial impressions, provides for the easy insertion of the denture, and that models from such impressions should be used untrimmed. This teaching seems clearly erroneous, because in inserting partial dentures certain movements (tilting, springing, etc.), obviate the use of the file to a great extent, and plates thus inserted are frequently aided in their retention.

The hardness of modelling composition is apt to produce a difficulty where there is much excess. The excess overflowing the rim of the tray may push out of position the buccal and labial muscles, causing them to drag with them the membrane which unites the cheek and gums, and thus prevent a close fit at the border of the plate. To avoid this the impression should be withdrawn before fully pressed to place, and after being slightly chilled, the excess cut away by a sharp razor, then re-warmed and returned to the mouth. The posterior excess should also be removed.

I am satisfied that more than half the failures with it arise from its being removed too early from the mouth, and that the instructions accompanying it are mainly responsible for this. The time required by the hard kind is less than by the soft, but is still at least four minutes in edentulous arches, and about seven where teeth remain; varying, of course, with the quantity used, etc. This may be lessened by injecting cold water, without danger of warpage. The cheek shouid be distented by the operator's finger so as to hold the water.

10th. To test warpage sulphuric ether was sprayed on a rope about an inch thick, and a foot long, without perceptibly warping it.

Where strong compression is required to equalize the pressure of a full plate on an irregularly hard and soft mouth, this composition used hard and with strong pressure is unparalleled as an impression material.

With regard to its ulterior uses, the writer has found it well adapted for duplicating models, but his experience with it for base plates and articulating impressions has been decidedly unfavorable.

In summing up it may be said of this material that it requires more skill and acquaintance with it to take a *correct* impression than either plaster or wax, but if understood is very useful and nice to use. Its elasticity is valuable where undercuts are slight; but its ductilily contra-indicates it where they are decided. Its hardness recommends it where strong compression is required. Its slight shrinkage in cooling is useful in counteracting the expansion of plaster. Trays used with it should have flanges suited in height to the mouth, and should extend far back. The composition should be made to firmly adhere to the tray; surplus material should be avoided, and if present removed by a razor. Never should the impression be finally removed till thoroughly cool.

The harder material proved itself in these experiments to be the better in almost every instance.—*Ohio State Journal.*

### Bacillus Culture.

In his lately published report to the Local Government Board on the "Relations of Septic to Pathogenic Organisms," Dr. Klein gives us ample material for reflection on this subject generally. The practical outcome of this paper is to throw doubt on M. Pasteur's statement that a culture of bacillus anthracis which has become inactive on sheep always yields farther cultures, which, although full of the typical anthrax bacilli, are, nevertheless, no longer pathogenic. By a series of experiments, Dr. Klein claims to have proved that there is no evidence whatever that a harmless micro-organism can be changed into a harmful one, or *vice versa,* and he believes that pathogenic bacteria are virulent *ab initio.* The experiments which have led him to this conclusion are given in detail, and are well worthy the perusal of all who would form clear ideas on this subject.

The fact of the cultivated bacillus affecting different species of animals in various ways, Dr. Klein attributes to varying conditions of growth. High temperatures, or a different soil, or other conditions may cause the bacilli, although remaining themselves the same, as can be experimentally shown, "to embody or appropriate, chemically or otherwise, some new or different substance, which produces the alteration for a particular species of animals." If Dr. Klein's experiments are to be regarded as having satisfactorily contradicted those of M. Pasteur with respect to the attenuation of the anthrax virus, their practical influence, even though exerted in a negative direction, can not fail to be very great. For it is on his results with the anthrax poison that M. Pasteur has built his hopes, and those of so many others, for the day of preventive inoculation against many of the infective diseases of mankind.

Dr. Klein completely denies Buchner's statement that the hay bacillus can be transformed by various cultivations into the anthrax bacillus, and that the latter may be made to assume the harmless properties of the former, supporting his denial by detailed experiments, and showing apparently good reasons, from his own experience, for believing that in Buchner's observations the culture of hay bacillus must have been accidentally contaminated by anthrax spores.

One more important observation is made by Dr. Klein touching the method of cultivation of the bacilli, which, as much as anything else in his suggestive and valuable paper, serves to point the moral with which the present article begins. He argues that the culture of bacilli on solid material, rich in gelatine, does not exclude the possibility, or even, in some cases the probability of some poisonous material being retained, arising from the original source, and apart from the organisms themselves. In some instances he gives ample evidence that this is probably the case.

It would seem from these considerations that there is good reason for medical men to hesitate at present in the formulation of their creeds and doctrines in the matter of the ætiology of infectious diseases, for when men, like Klein and Pasteur and Buchner and many others are at varience at the outset of inquiry, it is purile for the most practical physicians to try conclusions with one another.—*London Medical Times.*

